# Psychometric properties of the Cognitive Functioning Self-Assessment Scale in community-dwelling adults: A cross-sectional online survey

**DOI:** 10.3389/fpsyg.2023.1122198

**Published:** 2023-02-23

**Authors:** Krista Ching Wai Chung, Sujin Nam, Sha Li, Heidi Sze Lok Fan, Janet Yuen Ha Wong, Jojo Yan Yan Kwok, Hugh Davies, Lixi Huang, Maria Antonietta Annunziata, Daniel Yee Tak Fong

**Affiliations:** ^1^School of Nursing, Li Ka Shing Faculty of Medicine, The University of Hong Kong, Pokfulam, Hong Kong SAR, China; ^2^School of Nursing, Nanjing Medical University, Nanjing, China; ^3^School of Nursing, Faculty of Health and Social Development, University of British Columbia, Kelowna, BC, Canada; ^4^School of Nursing and Health Studies, Hong Kong Metropolitan University, Kowloon, Hong Kong SAR, China; ^5^School of Population and Public Health, University of British Columbia, Vancouver, BC, Canada; ^6^Department of Mechanical Engineering, Faculty of Engineering, The University of Hong Kong, Pokfulam, Hong Kong SAR, China; ^7^Unit of Oncological Psychology, Centro di Riferimento Oncologico di Aviano (CRO) IRCCS, Aviano, Pordenone, Italy

**Keywords:** Chinese, Cognitive Functioning Self-Assessment Scale, online survey, reliability, validation, cognitive disorders, mild cognitive impairment, dementia

## Abstract

**Background:**

The Cognitive Functioning Self-Assessment Scale (CFSS) was developed to allow the self-assessment of perceived cognitive functioning. It has been tested with satisfactory reliability and validity but was not available for the Chinese population. This study aimed to adapt and validate the Chinese version of the CFSS for community-dwelling adults.

**Methods:**

A cross-sectional study of a sample of 1,002 Chinese adults from the general population was conducted online (July–August 2022). The Chinese version of the CFSS with 18 items was created through translation, cognitive debriefing, and psychometric evaluation. Exploratory factor analysis (EFA) was performed on the first half of the randomly split sample. A model derived from EFA was confirmed by confirmatory factor analysis (CFA) in the second half of the sample. The model fits were further evaluated with and without subgrouping by age, gender, and education level. Internal consistency was assessed using Cronbach’s alpha (α) and McDonald’s omega hierarchical coefficients (ωH).

**Results:**

EFA (*n* = 460) revealed a three-factor solution, including spatio-temporal orientation, attention, and memory, which explained 51% of the total variance. The second-order CFA (*n* = 510) demonstrated a good fit: CFI = 0.951, TLI = 0.944, RMSEA = 0.054, SRMR = 0.040. A second-order multiple-group analysis showed that the structure was invariant by age, gender, and education level. The total CFSS score was significantly associated with the Chinese version of the nine-item Patient Health Questionnaire (*r* = 0.56, *p*< 0.001) and the Chinese version of the seven-item General Anxiety Disorder (*r* = 0.53, *p*<0.001). The internal consistency reliability was satisfactory, with *α* = 0.94 and ωH = 0.84. The item-total correlation coefficients ranged from 0.58 to 0.72.

**Conclusion:**

The Chinese version of the CFSS possesses good item characteristics, satisfactory validity, and reliability for assessing self-reported cognitive functioning among community-dwelling adults. It is a feasible and appropriate self-assessment instrument to examine cognitive functioning in the Hong Kong Chinese population, which contributes to monitoring and developing strategies to prevent and manage cognitive impairment and disorders for the public’s cognitive health and well-being.

## 1. Introduction

Cognitive functioning, which represents the mental process encompassing memory, attention, executive functions, language, and perception, is a vital part of an individual’s life, especially concerning independence in social and work areas (Annunziata et al., 2012). Mild cognitive impairment (MCI) often acts as a preclinical stage of dementia (Morris, 2005). Both MCI and dementia can have varied etiopathogenesis, such as neurodegenerative, vascular, or mixed (Costanza et al., 2012). In particular, MCI could be distinctively classified as (a) amnestic or non-amnestic based on the presence of memory impairment, and (b) single or multiple domains based on whether non-memory cognitive domains (e.g., attention, executive functioning, and language ability) are also involved (Petersen, 2004). Similar to multiple-domain amnestic MCI, dementia can affect both memory and non-memory cognitive domains (World Health Organization, 2022). However, progressing from MCI to dementia is beyond the expected consequences of normal aging (World Health Organization, 2022) to the extent of interfering with functional abilities (e.g., social and occupational functioning) (Hugo and Ganguli, 2014). Dementia may often be associated with mood and behavioral changes and can manifest in different forms such as Alzheimer’s disease (AD), vascular dementia, dementia with Lewy bodies, frontotemporal dementia, and mixed forms (World Health Organization, 2022). While vascular dementia can be attributable to neuronal loss related to ischemic brain damage including stroke (Uwagbai and Kalish, 2022), other forms of dementia may involve neuronal loss due to an abnormal deposition of native proteins in the brain (Emmady et al., 2022).

The estimated global prevalence of MCI was over 15% among community-dwelling adults aged 50 and above in a recent systematic review and meta-analysis (Bai et al., 2022). Approximately 10 million new cases of dementia are reported annually worldwide (World Health Organization, 2022). In 2019, the estimated total global societal cost associated with dementia was USD 1.3 trillion, which may exceed USD 2.8 trillion by 2030 (World Health Organization, 2022). Thus, there has been an increasing economic burden of dementia globally. The recent Coronavirus disease 2019 (COVID-19) pandemic has impaired the cognitive function due to the social distancing measures (Fiorenzato et al., 2021). In Hong Kong, the suspension of dementia daycare services has resulted in deteriorated cognitive functioning among dementia patients (Wong et al., 2021). Given the pandemic’s detrimental impacts on cognitive functioning both globally and locally, public health concerns and the importance of assessing cognitive functioning are growing.

Notably, while cognitive impairment can accompany psychiatric complications, psychiatric symptoms, including depression and anxiety, may also increase cognitive decline. Specifically, changes in visual and spatial working memory are more likely to be associated with psychiatric symptoms (Singham et al., 2022). A population-based cohort study found that anxiety symptoms were associated with worsened processing speed and cognitive impairment (Gulpers et al., 2022). Furthermore, anxiety disorders, especially agoraphobia, are associated with worsened cognitive functioning (e.g., executive functioning, memory, and processing speed) and cognitive impairment (Gulpers et al., 2022). The presence of depressive symptoms is also associated with cognitive impairment, which may be predictive of early- and late-onset depression (Jamieson et al., 2019). In the context of Hong Kong, a recent study involving advanced lung cancer patients found that anxiety was a significant factor for their perceived cognitive impairment in terms of attention in 35.4% of patients and memory in 58.4% of patients (Takemura et al., 2022). Another study of the general elderly found that depressive and anxiety symptoms were negatively associated with lateral prefrontal cortex function during cognitive control performance (Yeung et al., 2021). Thus, these psychiatric complications can exacerbate cognitive symptoms. Moreover, anxiety and depression, among other psychiatric symptoms, are the most frequent in the early stages of cognitive disorders. The neurological psychiatric interactions may increase the risk of suicide (Costanza et al., 2015, 2020). Thus, screening of MCI in the general population may indeed be the first essential step to prevent psychiatric complications, suicide risk, or even an exacerbation of impaired cognition.

Early assessment of MCI is the key to preventing cognitive impairment and managing cognitive disorders in both community and clinical settings. Various cognitive functioning assessments are available for the Chinese population. Several screening instruments have been developed for the Chinese with mild cognitive impairment in Hong Kong. For instance, the Hong Kong Montreal Cognitive Assessment (HK-MoCA), which takes approximately 10 minutes, is administered by interviewing patients with cerebral small vessel diseases (Wong et al., 2009). Subsequently, it has also been tested among patients with stroke and transient ischemic attacks to administer a shorter interview version of the HK-MoCA *via* telephone interviews (Wong et al., 2015). Moreover, the Cantonese version of the Mini-Mental State Examination (C-MMSE) for the elderly and patients with dementia (Chiu, 1994) and the Hong Kong version of the Oxford Cognitive Screen (HK-OCS) for the stroke population (Kong et al., 2016) are also available. However, most of these were developed primarily to screen individuals with MCI and require an interviewer to administer them. Apart from objective diagnostic criteria, subjective cognitive impairment may also be crucial in the clinical diagnosis of MCI (Bradfield, 2023). To our knowledge, a self-reported instrument to assess the level of perceived cognitive functioning of the Hong Kong Chinese population has not been available. Considering the current pandemic’s progression and future pandemic preparedness, it is imperative to address the public’s cognitive issues in a timely and appropriate manner. The administration of self-reported instruments in lieu of in-person interviews could reduce the burden of using devices and equipment for instrument administration and minimize face-to-face contact. This mode of administration without geographical or temporal limitations may be necessary, especially in the pandemic situation.

Compared to cognitively unimpaired individuals without subjective cognitive decline (SCD), those with SCD had a higher risk of cognitive decline and dementia (Jessen et al., 2020). The 18-item Cognitive Functioning Self-Assessment Scale (CFSS) can be widely used as a self-reported instrument to assess cognitive functioning (Annunziata et al., 2012, 2018). This instrument would be beneficial when assessing cognitive functioning in a community setting in terms of the quality-of-life concept, which is usually neglected, generating differential diagnoses by incorporating mood disorders and neuropsychological issues, and providing patients’ perceived cognitive functioning (Annunziata et al., 2018). It comprises 18 items that cover the cognitive domains of attention, memory, and spatio-temporal orientation conceptually (Annunziata et al., 2018). This scale was tested on 282 patients who visited general practitioners in Italy. It has a one-dimensional factorial structure with an internal consistency of 0.86 (Annunziata et al., 2012). Psychometric properties were further examined with a similar group of patients, with good internal reliability of 0.88 and satisfactory test–retest stability of 0.79 (Annunziata et al., 2018). It has also demonstrated expected associations with depression, anxiety, age, and education (Annunziata et al., 2018). Moreover, in 198 long-term lymphoma survivors, the Italian CFSS showed a significantly positive association with the number of impaired and borderline performances, but a negative association with the number of normal performances and quality-of-life (Muzzatti et al., 2021). Despite its potential merits, CFSS has not been tested in a community setting and has not been available for the Chinese population.

In response to the lack of a self-reported instrument for assessing cognitive functioning in a Chinese community setting, this study aims to adapt and evaluate the psychometric performance of the Chinese version of the CFSS with the evaluation of factorial structure and invariance in the general Chinese population. The Chinese CFSS would facilitate the efficient assessment of perceived cognitive functioning and allow healthcare providers and policymakers to devise compelling strategies and interventions to prevent and manage the public’s cognitive impairment and disorders in a timely and cost-effective manner.

## 2. Methods

### 2.1. Study design and participants

This cross-sectional online study was conducted among the general population in Hong Kong from July to August 2022. The inclusion criteria were (a) residing in Hong Kong, (b) being 18 years or older, and (c) being literate in traditional Chinese. Those who did not agree to participate were excluded. The sample size calculation was based on an assessment of the factor structure in two random halves. The literature suggested 10–20 subjects per item for the respective factor analysis (Hair et al., 2019). For the 18-item CFSS, 360 subjects for a factor analysis would be needed. Thus, 720 participants were required. Allowing sizeable incomplete responses in an online survey, we targeted 1,000 participants. To avoid social contact during the pandemic, we sought a double opt-in online panel service to recruit participants who met the eligibility criteria by email and text messages. The respondents were invited to complete an online questionnaire voluntarily on their electronic devices and a total of 1,002 eligible responses were obtained.

### 2.2. Study procedures

#### 2.2.1. Forward–backward translation

According to the translation guidelines of the International Society for Pharmacoeconomics and Outcomes Research (ISPOR) Principles of Good Practice (Wild et al., 2005), the English version of the CFSS provided by the developer was independently translated into traditional Chinese, by two bilingual Cantonese-speaking Registered Nurses (RNs). RNs who possessed linguistic ability and adequate clinical knowledge regarding discerning clients’ cognitive functioning levels using screening instruments independently performed the translation. The differences between the two forward-translated Chinese versions were discussed in a reconciliation meeting involving the two translators and a researcher with prior experience in the cultural adaptation of patient-reported outcomes. The consensus Chinese version was back-translated into English by another bilingual RN, who was unaware of the CFSS. Amendments to the pre-final version of the translated CFSS were made based on comparisons between the two English versions during the back-translation review.

#### 2.2.2. Cognitive debriefing

The pre-final version of the CFSS was assessed by a cognitive debriefing interview with five individuals to ensure comprehension, interpretation, and cultural relevance among the target population. Interviewees were asked to rate the acceptability of the length on a five-point Likert scale (0, representing “very short” to 4, representing “very long”). The duration of the completion of the questionnaire was recorded. Interviewees were also asked to indicate the overall relevance and clarity of the CFSS on a five-point Likert scale (0, representing “very low” to 4, representing “very high”). One minor amendment was made to item 8, “I find it difficult to remember recent information (e.g., a person, place, or product name),” in which the example of a phone number was excluded from the item. As smartphone usage has increased to 92.9% in 2021 in Hong Kong (Census and Statistics Department, 2022b). Its phone book function has been widely used without the need to memorize phone numbers.

### 2.3. Psychometric evaluation

#### 2.3.1. Measurements

##### 2.3.1.1. The Cognitive Functioning Self-Assessment Scale

The CFSS is a self-administered questionnaire that assesses cognitive functioning and it was originally validated within an Italian population (Annunziata et al., 2012, 2018). It comprises 18 statements about daily life activities involving cognitive domains (e.g., attention, memory, and spatio-temporal orientation). Respondents rated the frequency of performing these activities in the past year on a five-point Likert scale (1, representing “never” to 5, representing “always”). This scale has one dimension (Annunziata et al., 2012). The total score was calculated as the mean of the summed scores, ranging from 1 to 5. A higher total score indicates more aggravated self-perceived cognitive functioning. In the original study, Cronbach’s alpha value and the test–retest reliability value of the scale were 0.88 and 0.79, respectively (Annunziata et al., 2018).

##### 2.3.1.2. The Patient Health Questionnaire-9 (PHQ-9)

The Patient Health Questionnaire-9 (PHQ-9) is a nine-item self-administered screening scale for depressive symptoms (Yu et al., 2012). It assesses the frequency of depressive symptoms within the past 2 weeks on a four-point Likert scale (0, representing “not at all” to 3, representing “nearly every day”). The total score ranges from 0 to 27. The severity of depressive symptoms is categorized into five groups: minimal (0–4), mild (5–9), moderate (10–14), moderately severe (15–19), and severe (20 or higher). In the validation study among the Chinese population, the internal consistency and test–retest reliability were 0.82 and 0.76, respectively (Yu et al., 2012).

##### 2.3.1.3. The Generalized Anxiety Disorder-7

The Generalized Anxiety Disorder-7 (GAD-7) is a seven-item self-administered screening instrument. It assesses the frequency of anxiety symptoms within the past 2 weeks. Scores of 0, 1, 2, and 3 are assigned to the frequency categories of “not at all,” “several days,” “more than half the days,” and “nearly every day.” The total score ranges from 0 to 21. A total score of 5, 10, and 15 indicate mild, moderate, and severe anxiety, respectively (Tong et al., 2016). In a validation study among Chinese epilepsy outpatients, Cronbach’s alpha coefficient for this scale was 0.90, and the test–retest reliability was 0.86 (Tong et al., 2016).

##### 2.3.1.4. Demographics and work-related information

Demographics, including age, gender, education level, marital status, employment status, monthly household income (Hong Kong dollars), and district of residence, were assessed.

#### 2.3.2. Validity check question

To identify inconsistent responses, that is, those that were given without paying attention to the questions, we inserted a validity check question in a random position in the questionnaire. The validity check question was randomly selected from three questions: “Please select ‘sometimes’ as your answer of choice to show that you are paying attention to the questions,” with “sometimes” replaced by “never” and “most or all of the time.” Individuals who did not respond correctly to the validity check question were excluded from the analysis.

#### 2.3.3. Statistical analysis

All statistical tests were conducted using RStudio version (4.2.1) with a nominal significance level of 0.05. During the data-cleaning procedure, 32 inconsistent responses were detected and excluded before data analysis. Sample characteristics were summarized using descriptive statistics, including mean, standard deviations (*SDs*), skewness, and kurtosis. With a sample size >300, a substantially non-normal distribution can be identified by either an absolute skewness value >2 or an absolute kurtosis value >7 (Curran et al., 1996).

To explore the structural validity of the Chinese version of the CFSS, the sample was randomly divided into two halves. The first half (*n* = 460) was used in exploratory factor analysis (EFA) as a training set. Before conducting EFA, sampling adequacy was evaluated using Bartlett’s test of sphericity and the Kaiser–Meyer–Olkin (KMO) test. Bartlett’s test of sphericity should be significant (*p*-value < 0.05), and a KMO value higher than 0.50 is required (Hair et al., 2019). The EFA was conducted with oblimin rotation using the ‘psych’ package. Parallel analysis was applied to determine the number of factor loadings (Hair et al., 2019). A factor loading estimate of a minimum of 0.30 is required to adequately interpret the factor structure (Hair et al., 2019). The other half of the sample (*n* = 510) served as the validation set for the model derived from EFA.

Confirmatory factor analysis (CFA) was performed using the “lavaan” package. The goodness-of-fit was assessed based on the root mean square error of approximation (RMSEA), standardized root mean square residual (SRMR), comparative fit index (CFI), and the Tucker–Lewis Index (TLI). Specifically, CFA models are considered adequately fitted when the *p*-values are significant for Chi-square (Hair et al., 2019), RMSEA and SRMR are lower than 0.08 (Hooper et al., 2008), and CFI and TLI are greater than 0.90 (Hu and Bentler, 1999). Furthermore, a model that yields the smallest Akaike information criterion (AIC) or Bayesian information criterion (BIC) value is preferred (Hair et al., 2019). In a second-order CFA model, when a second-order factor contributes to an underlying association between first-order factors, the first-order factors could act as latent indicators of the latent second-order factor. In other words, a first-order factor is regarded as an indicator instead of an observed outcome while the endogenous factors have their residual variances (University of California, Los Angeles, 2021). To examine whether the first-order factors can be explained by a second-order factor, testing of a hierarchical model was conducted.

Based on the association between CFSS and age and education level (Annunziata et al., 2018) and a significant difference by gender in terms of CFSS score (Annunziata et al., 2012), we further evaluated the second-order multi-group CFA models to test equality in factor loadings for the hierarchical model by subgroups. Given a peak of cognitive performance at an approximate age of 35 (Strittmatter et al., 2020), 54% of the local population being female (Census and Statistics Department, 2022a), and an increased proportion of individuals attaining post-secondary education in Hong Kong (Census and Statistics Department, 2021a), the second-order multi-group analyses were conducted using the established models for the subgroups to examine variances by age, gender, and education level.

Comparisons were made between the models with and without equality constraints. A non-significant difference (*p*-value > 0.05) in the Chi-square values indicates equal factor loadings across subgroups. A non-significant difference between hierarchical models with and without constraints in a Chi-square difference test indicates that the path estimates are the same between the subgroups and that moderation is not indicated (Hair et al., 2019). Moreover, the relations to other variables were estimated to evaluate the validity of test scores with its proposed usage (American Educational Research Association; American Psychological Association; National Council on Measurement in Education, 2014). The relation of the CFSS score to external variables was measured based on Pearson’s correlation coefficients between the total CFSS score and (a) depressive symptoms using the PHQ-9 and (b) anxiety symptoms using the GAD-7. It was hypothesized that a higher total CFSS score would be positively associated with depressive and anxiety symptoms.

Internal consistency was measured using Cronbach’s alpha and McDonald’s omega hierarchical coefficients. Cronbach’s alpha is a measure of reliability, with values of 0.70 considered acceptable (Hair et al., 2019). The corrected item-total correlations were also measured, and a minimum level of 0.30 is required to ensure that the items were distinctive (Streiner et al., 2015). McDonald’s omega hierarchical coefficient, assessing the percentage of variance that could be explained by the general factor with all other dimension-specific factors under control (Streiner et al., 2015), was calculated. When the ideal value of the omega hierarchical coefficient of >0.80 is achieved, uni-dimensionality can be considered (Pirani et al., 2021).

### 2.4. Ethical approval

The study was conducted in accordance with the principles of the Declaration of Helsinki and approved by the Institutional Review Board of the University of Hong Kong/Hospital Authority Hong Kong West Cluster (HKU/HA HKW IRB). Before voluntarily participating in this study, the subjects were provided with information on the research purpose and characteristics. Informed consent was obtained by checking the selection box. The anonymity and privacy of the data collected from the participants were maintained.

## 3. Results

### 3.1. Demographic characteristics

After removing 32 inconsistent responses from the dataset, 970 of the 1,002 participants were included in the study. The demographic information of all the participants (n = 970) is summarized in Table 1. With a mean age of 39.7 (*SD* = 13.8) years, 56% of participants were female. Nearly 90% of the participants worked (*n* = 864). Approximately 70% of the participants attained an education level of university or above (*n* = 664). More than one-third of the participants (36.6%) earned equal to or greater than 50,000 Hong Kong dollars per month. Approximately half of them were married (48.4%) and lived in the New Territories area of Hong Kong (47.8%).

**Table 1:** Demographic information about the participants (*n* = 970).

Characteristics	Categories	*n* (%)/Mean ± SD
Age (years)		39.7 ± 13.8
	18–29	289 (30.0)	30–39	211 (21.8)	40–49	192 (19.8)	50–59	189 (19.5)	≥60	89 (9.2)
Gender
	Male	425 (44.0)	Female	545 (56.0)
Education level
	Primary school or below	14 (1.4)	Secondary school, diploma or associate degree	292 (30.1)	University or above	664 (68.5)
Marital status
	Single	444 (45.8)	Married	469 (48.4)	Divorced/separated	20 (2.1)	Widowed	8 (0.8)	Cohabitation	29 (3.0)
Employment status
	Employed or self-employed	864 (89.1)	Employer	11 (1.1)	Housewife, unemployed, retired	57 (5.9)	Student	38 (3.9)
Monthly household income (HK $)
	<5,000	10 (1.0)	5,000–19,999	86 (8.9)	20,000–34,999	241 (24.8)	35,000–49,999	278 (28.7)	≥50,000	355 (36.6)
District of residence
	Hong Kong Island	227 (23.4)	Kowloon	279 (28.8)	New Territories	464 (47.8)

### 3.2. Cognitive debriefing

The pre-final version of the CFSS was completed by five participants rating on a five-point Likert scale. It took an average of 3.7 minutes (*SD* = 2.3) for the participants to complete the CFSS. Apart from one interviewee who deemed the questionnaire long, all others perceived it to be acceptable in length. The mean overall relevance score was 2.8 (*SD* = 0.4), indicating a moderate level. The mean overall clarity score was 2.8 (*SD* = 0.4), indicating a level between moderate and high.

### 3.3. Psychometric properties

Table 2 shows the characteristics (e.g., mean, *SDs*, the proportion of frequency, skewness, and kurtosis) of the CFSS with 18 items among all participants (*n* = 970). Substantially non-normal distributions were not indicated, with the items’ skewness and kurtosis values being 0.12 and 0.83. The total mean score of the CFSS was 2.46 (*SD* = 0.61), ranging from 1 to 5. This scale has a single dimension and three subscales, including spatio-temporal orientation, attention, and memory. The first subscale, “spatio-temporal orientation”, included nine items (items 3, 4, 7, 9, 10, 13, 14, 17, and 18). The second subscale, “attention”, included five items (items 1, 2, 6, 8, and 12). The third subscale, “memory”, included four items (items 5, 11, 15, and 16). Subscale averages were 2.35 (*SD* = 0.65) for the first factor, 2.60 (*SD* = 0.67) for the second factor, and 2.53 (*SD* = 0.67) for the third factor (Table 2).

**Table 2:** Item characteristics (*n* = 970).

Item no.	Contents (subscale)	Mean (SD)	Never *n* (%)	Almost never*n* (%)	Sometimes *n* (%)	Almost always *n* (%)	Always *n* (%)	Skewness	Kurtosis	Corrected item-total correlation
1	Lack of concentration (Attention)	2.65 (0.86)	97 (10.0%)	280 (28.9%)	478 (49.3%)	98 (10.1%)	17 (1.8%)	−0.04	0.08	0.67
2	Absent-mindedness (Attention)	2.69 (0.84)	80 (8.2%)	281 (29.0%)	492 (50.7%)	95 (9.8%)	22 (2.3%)	0.04	0.31	0.72
3	Difficulty in performing two tasks simultaneously (Spatio-temporal orientation)	2.30 (0.92)	196 (20.2%)	388 (40.0%)	306 (31.5%)	63 (6.5%)	17 (1.8%)	0.42	−0.03	0.64
4	Difficulty in performing mental calculation (Spatio-temporal orientation)	2.48 (0.97)	156 (16.1%)	341 (35.2%)	347 (35.8%)	100 (10.3%)	26 (2.7%)	0.30	−0.23	0.63
5	Tip of the tongue phenomenon (Memory)	2.61 (0.84)	104 (10.7%)	276 (28.5%)	506 (52.2%)	65 (6.7%)	19 (2.0%)	−0.05	0.34	0.70
6	Absent-mindedness during intellectual/cognitive activities (Attention)	2.57 (0.86)	116 (12.0%)	292 (30.1%)	466 (48.0%)	82 (8.5%)	14 (1.4%)	−0.05	−0.02	0.67
7	Difficulty in organizing extra-routine activities (Spatio-temporal orientation)	2.27 (0.92)	202 (20.8%)	399 (41.1%)	292 (30.1%)	59 (6.1%)	18 (1.9%)	0.48	0.07	0.63
8	Difficulty in recalling recent information (Attention)	2.51 (0.87)	126 (13.0%)	324 (33.4%)	431 (44.4%)	73 (7.5%)	16 (1.6%)	0.08	−0.01	0.70
9	Difficulty in recalling old information (Spatio-temporal orientation)	2.51 (0.89)	133 (13.7%)	327 (33.7%)	409 (42.2%)	87 (9.0%)	14 (1.4%)	0.08	−0.19	0.67
10	Difficulty in recalling autobiographical events (Spatio-temporal orientation)	2.50 (0.92)	134 (13.8%)	349 (36.0%)	376 (38.8%)	88 (9.1%)	23 (2.4%)	0.27	−0.06	0.69
11	Forgetfulness (Memory)	2.48 (0.81)	106 (10.9%)	372 (38.4%)	425 (43.8%)	56 (5.8%)	11 (1.1%)	0.10	0.16	0.58
12	Lack of concentration while reading (Attention)	2.55 (0.88)	117 (12.1%)	317 (32.7%)	434 (44.7%)	85 (8.8%)	17 (1.8%)	0.08	−0.02	0.65
13	Lack of motor coordination (Spatio-temporal orientation)	2.19 (0.92)	237 (24.4%)	390 (40.2%)	279 (28.8%)	49 (5.1%)	15 (1.5%)	0.49	0.02	0.62
14	Slowness in the execution of movements (Spatio-temporal orientation)	2.39 (0.90)	165 (17.0%)	361 (37.2%)	360 (37.1%)	70 (7.2%)	14 (1.4%)	0.23	−0.18	0.68
15	Difficulty in finding the appropriate words (Memory)	2.53 (0.86)	115 (11.9%)	336 (34.6%)	429 (44.2%)	73 (7.5%)	17 (1.8%)	0.12	0.09	0.71
16	Use of periphrases or generic terms instead of specific words (Memory)	2.51 (0.87)	124 (12.8%)	323 (33.3%)	441 (45.5%)	64 (6.6%)	18 (1.9%)	0.10	0.12	0.69
17	Difficulty in spatial orientation (Spatio-temporal orientation)	2.27 (0.92)	205 (21.1%)	391 (40.3%)	304 (31.3%)	51 (5.3%)	19 (2.0%)	0.47	0.12	0.62
18	Difficulty in temporal orientation (Spatio-temporal orientation)	2.26 (0.94)	231 (23.8%)	350 (36.1%)	317 (32.7%)	54 (5.6%)	18 (1.9%)	0.40	−0.15	0.65
		**Mean (SD)**	**Min**	**Max**	**Floor (%)**	**Ceiling (%)**		**Skewness**	**Kurtosis**	
	Total score	2.46 (0.61)	1	5	1.5	0.1		0.12	0.83	
	Spatio-temporal orientation	2.35 (0.65)	1	5	2.6	0.2		0.29	0.64	
	Attention	2.60 (0.67)	1	5	3.1	0.4		−0.04	0.63	
	Memory	2.53 (0.67)	1	5	3.8	0.3		−0.04	0.63	

SD, standard deviation; Floor (%), percentage of respondents with the lowest possible score; Ceiling (%), percentage of respondents with the highest possible score.

**Figure 1:**
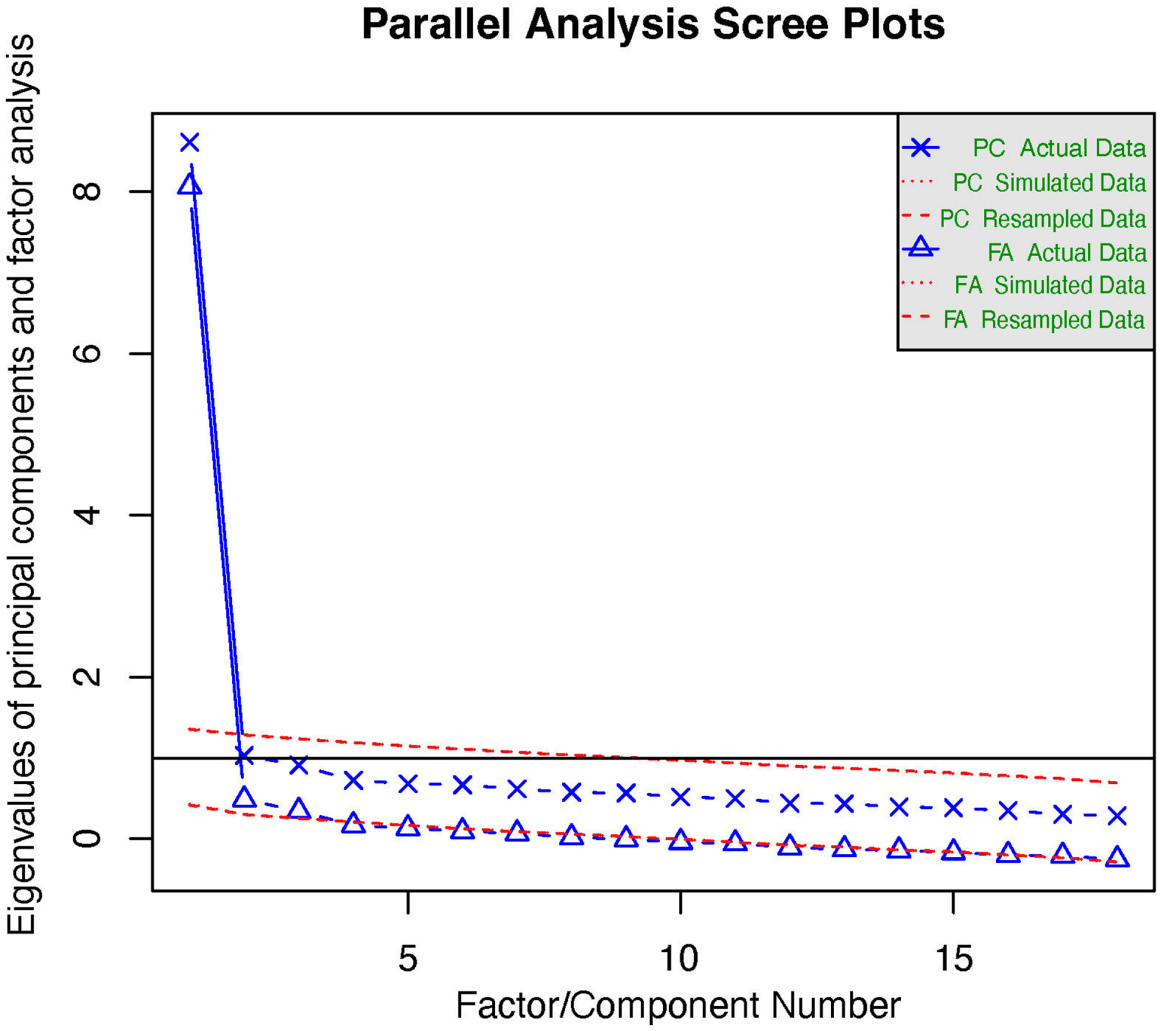
Scree plot of the Chinese version of the CFSS.

Using the first half of the data (*n* = 460), an EFA test with an oblimin rotation yielded a three-factor structure. Sampling adequacy was indicated by a significant Bartlett’s test of sphericity [χ^2^ = 4069.353, degrees of freedom (df) = 153, *p* < 0.001] and a KMO value of 0.96, over 0.50. To examine structural validity using EFA, the three-factor structure based on the results of the parallel analysis in Figure 1 explained 51% of the total variance (Table 3). All items had factor loading estimates of >0.3. Specifically, factor loading estimates ranging between 0.32 and 0.74 were observed in the first subscale. The factor loading estimates ranged between 0.34 and 0.83, and between 0.45 and 0.81 in the second and third subscales, respectively.

**Table 3:** Factor loading values of an exploratory factor analysis after the oblimin rotation (*n* = 460).

Contents (Item no.)	Factor 1: Spatio-temporal orientation	Factor 2: Attention	Factor 3: Memory	Cronbach’s alpha	Total Cronbach’s alpha
Difficulty in performing two tasks simultaneously (3)	0.49			0.88	0.94
Difficulty in performing mental calculation (4)	0.51				
Difficulty in organizing extra-routine activities (7)	0.39				
Difficulty in recalling old information (9)	0.47				
Difficulty in recalling autobiographical events (10)	0.32				
Lack of motor coordination (13)	0.64				
Slowness in the execution of movements (14)	0.58				
Difficulty in spatial orientation (17)	0.74				
Difficulty in temporal orientation (18)	0.42				
Lack of concentration (1)		0.66		0.85	
Absent-mindedness (2)		0.83			
Absent-mindedness during intellectual/cognitive activities (6)		0.70			
Difficulty in recalling recent information (8)		0.34			
Lack of concentration while reading (12)		0.50			
Tip of the tongue phenomenon (5)			0.70	0.83	
Forgetfulness (11)			0.45		
Difficulty in finding the appropriate words (15)			0.76		
Use of periphrases or generic terms instead of specific words (16)			0.81		
Variance explained (%)	19.0%	16.0%	16.0%		
Total variance explained (%)	51.0%				

**Figure 2:**
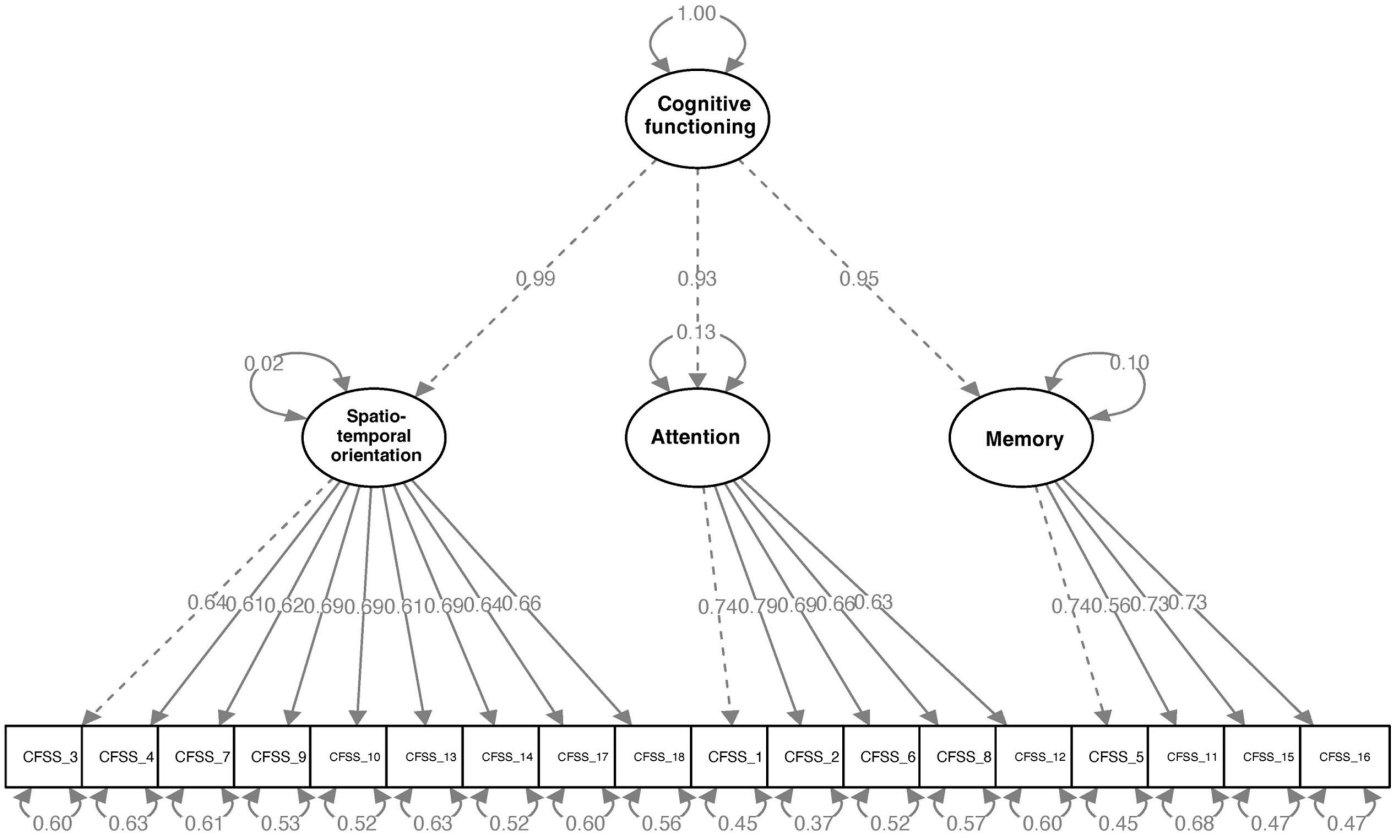
Standardized estimates of the confirmatory factor analysis of the CFSS.

With the second half of the data (*n* = 510), the second-order CFA was applied to confirm the proposed model based on the entire single latent construct in the EFA results. Our hierarchical model in which a second-order factor explained the correlations among three first-order factors. The second-order CFA results provided a good fit: χ^2^ = 335.075, *p* < 0.001, CFI = 0.951, TLI = 0.944, RMSEA = 0.054, SRMR = 0.040 (Table 4). The standardized factor loading values of the structure in the second-order CFA are presented in Figure 2. All standardized factor loading values were significant (*p* < 0.001) and greater than 0.50.

**Table 4:** Goodness-of-fit statistics for a second-order three-factor confirmatory factor analysis of the CFSS among Chinese adults (*n* = 510).

	*x*^2^ (df)	RMSEA (90%CI)	AIC	BIC	SRMR	CFI	TLI
**All participants**							
All (*n* = 510)	335.075 (134)	0.054 (0.047–0.062)	19670.034	19826.708	0.040	0.951	0.944
**By age**							
18–33 (*n* = 228)	264.007 (134)	0.065 (0.054–0.077)	9336.862	9463.748	0.058	0.925	0.914
33 > (*n* = 282)	249.279 (134)	0.055 (0.044–0.066)	10241.864	10376.614	0.046	0.953	0.946
**By gender**							
Female (*n* = 287)	272.697 (135)	0.060 (0.049–0.070)	11157.208	11354.820	0.040	0.942	0.935
Male (*n* = 223)	247.813 (135)	0.061 (0.049–0.073)	8589.950	8773.937	0.045	0.935	0.927
**By education level**							
Without university degrees (*n* = 163)	274.243 (134)	0.080 (0.067–0.094)	6325.058	6439.527	0.054	0.915	0.903
University and above (*n* = 347)	248.272 (134)	0.050 (0.049–0.059)	13349.713	13492.138	0.043	0.955	0.948
**Multi-group model to test factorial invariance**							
**Across age**							
Age (with no constraints)	513.286 (268)	0.060 (0.052–0.068)	19650.726	20116.511	0.049	0.941	0.933
Age (with equality constraints)	536.229 (283)	0.059 (0.052–0.067)	19643.668	20045.937	0.059	0.939	0.935
**Across gender**							
Gender (with no constraints)	520.511 (270)	0.060 (0.053–0.068)	19747.158	20204.474	0.042	0.939	0.931
Gender (with equality constraints)	532.993 (287)	0.058 (0.050–0.066)	19725.641	20110.972	0.049	0.941	0.937
**Across educational Level**							
Education level (with no constraints)	522.515 (268)	0.061 (0.053–0.069)	19746.771	20212.557	0.044	0.939	0.931
Education level (with equality constraints)	540.526 (283)	0.060 (0.052–0.067)	19734.783	20137.052	0.054	0.938	0.933

df, degrees of freedom; RMSEA, root mean squared error of approximation; CI, confidence interval; AIC, Akaike’s information criterion; BIC, Bayesian information criterion; SRMR, standardized root mean squared residual; CFI, comparative fit index; TLI, Tucker–Lewis index.

When sub-setting by age, gender, and education level groups, the respective hierarchical models were adequately fitted (Table 4). For different age groups, the model of adults aged between 18 and 33 years (*n* = 228) provided adequate goodness of fit (χ^2^ = 246.007, *p* < 0.001, CFI = 0.925, TLI = 0.914, RMSEA = 0.065, SRMR = 0.058). The results from the adults aged over 33 years (*n* = 282) were similar to those of adults aged between 18 and 33 years, which presented a satisfactory goodness of fit of the model (χ^2^ = 249.279, *p* < 0.001, CFI = 0.953, TLI = 0.946, RMSEA = 0.055, SRMR = 0.046). For gender groups, the model of female (*n* = 287) provided adequate goodness of fit (χ^2^ = 272.697, *p* < 0.001, CFI = 0.942, TLI = 0.935, RMSEA = 0.060, SRMR = 0.040). Similar to that of female, the model of male (*n* = 223) exhibited adequate goodness of fit (χ^2^ = 247.813, *p* < 0.001, CFI = 0.935, TLI = 0.927, RMSEA = 0.061, SRMR = 0.045). For different education levels, the model of participants without university degrees (n = 163) yielded an acceptable goodness of fit (χ^2^ = 274.243, *p* < 0.001, CFI = 0.915, TLI = 0.903, RMSEA = 0.080, SRMR = 0.054). The results from the subjects with an educational background of university and above (*n* = 347) were similar to the former and provided a satisfactory model (χ^2^ = 248.272, *p* < 0.001, CFI = 0.955, TLI = 0.948, RMSEA = 0.050, SRMR = 0.043). The second-order multi-group CFA analyses were performed to further examine factorial invariance. The difference in Chi-square values between the models with and without equality constraints across age (Δχ = 22.943, df = 15, *p* = 0.085), gender (Δχ = 12.483, df = 17, *p* = 0.077), and education levels (Δχ = 18.012, df = 15, *p* = 0.262) was insignificant. Therefore, the results demonstrated that the Chinese version of the CFSS has an identical structure across age, gender, and education levels.

Regarding the relation to other variables, the CFSS total score was significantly correlated with depressive symptoms assessed using the PHQ-9 (mean = 6.10, *SD* = 5.26) of 0.56 (*p* < 0.001). It was also significantly correlated with anxiety symptoms assessed using the GAD-7 (mean = 5.77, *SD* = 4.43) of 0.53 (*p* < 0.001) (Table 5). This demonstrated the sufficient convergent validity of the CFSS. With respect to internal consistency, the total Cronbach’s alpha coefficient was 0.94 (*n* = 970) (Table 3), which exceeds the minimum acceptable level of 0.70. The McDonald’s omega hierarchical coefficient of the CFSS total score was 0.84 (Table 5), which exceeds the ideal value of 0.80, and is considered unidimensional. The corrected item-total correlations of all items were > 0.30 (Table 2), which indicates that all items were correlated with the whole scale.

**Table 5:** Relation to other variables, including PHQ-9 and GAD-7, and reliability of the Chinese version of the CFSS (*n* = 510).

Relation to other variables: Pearson’s correlation (*r*)			Internal consistency: McDonald’s omega hierarchical (ωH)/McDonald’s omega (ω)
	PHQ-9	GAD-7	
CFSS total score	0.56^*^	0.53^*^	0.84
Spatio-temporal orientation	0.54^*^	0.50^*^	0.79
Attention	0.53^*^	0.50^*^	0.70
Memory	0.46^*^	0.46^*^	0.77
PHQ-9			0.92^†^
GAD-7			0.93^†^

PHQ-9, the 9-item Chinese Patient Health Questionnaire-9; GAD-7, the 7-item Chinese General Anxiety Disorder-7 (GAD-7); ^*^p < 0.001; ^†^McDonald’s omega.

## 4. Discussion

### 4.1. Summary of findings

We evaluated the Chinese CFSS, which is the first self-reported instrument for assessing perceived cognitive functioning among the general Chinese population in Hong Kong. This study provides evidence through robust testing of the psychometric properties of the Chinese CFSS. This involved a translation process conducted by healthcare professionals, particularly RNs, considering the clinical context. It also involved a cognitive debriefing with community-dwelling adults to expand from the clinical context to the community context. Our results suggest that the overall single dimension as well as the three subscales of spatio-temporal orientation, attention, and memory are reliable and valid for evaluating the cognitive functioning levels of Chinese community-dwelling individuals. The use of the self-reported instrument, the Chinese CFSS, would be advantageous for assessing perceived cognitive functioning to minimize face-to-face contact and reduce the burden of adopting devices and equipment for questionnaire administration in the infectious disease context.

### 4.2. Interpretation of findings

The Chinese CFSS possesses an essentially unidimensional structure, which aligns with the single-factor structure of the original CFSS identified using EFA (Annunziata et al., 2012). Indeed, the three-factor solution of the Chinese CFSS explained 51% of the total variance, which is substantially larger than the 31% of the original CFSS (Annunziata et al., 2012). The Chinese CFSS also has three subscales that were not verified in the original CFSS. These are the spatio-temporal orientation, attention, and memory subscales, which include nine, five, and four items, respectively. The structure of the second-order three-factor was confirmed in our second-order CFA based on independent data and was also invariant across sex and age groups. The same structure can be examined in the original CFSS and other language versions to foster collaborative research locally and globally for an improved understanding of cognitive functioning, thereby promoting the public’s cognitive health and well-being.

In the Hong Kong Chinese population, the total mean score of the CFSS was 2.46 (*SD* = 0.61), which was slightly higher than that in previous studies on patients receiving general medical services in Italy with a mean of 2.14 (*SD* = 0.47) (Annunziata et al., 2018), and cancer patients in Italy with a mean of 2.31 (*SD* = 0.63) (Muzzatti et al., 2021). Owing to limited research investigating cognitive functioning assessed using the CFSS worldwide (Annunziata et al., 2012, 2018), a direct comparison with our study might be impossible. Nevertheless, in comparison with the Italian study conducted before the outbreak of COVID-19, the relatively higher total score of the CFSS in this study might demonstrate more vulnerable cognitive functioning being affected by the COVID-19 pandemic among the Chinese population in Hong Kong. In addition to the total score, we identified the following subscales: spatio-temporal orientation (mean = 2.35, *SD* = 0.65), attention (mean = 2.60, *SD* = 0.67), and memory (mean = 2.53, *SD* = 0.67). According to a pandemic study in Italy, the COVID-19 lockdown negatively affected the general population’s cognitive functioning, including attention, executive functions, and temporal orientation (Fiorenzato et al., 2021). Thus, future studies investigating the factors associated with not only comprehensive impaired cognitive functioning but also more specific cognitive domains, including spatio-temporal orientation, attention, and memory, are needed to design effective intervention programs for the Chinese population in Hong Kong to address long-term responses to cognitive challenges during the pandemic.

The most salient point is the positive association between worse self-perceived cognitive functioning and psychiatric symptoms in community-dwelling adults while examining the validity of the CFSS in the current study. This finding is in line with the evidence from prior studies (Fung et al., 2018; Fiorenzato et al., 2021; Gulpers et al., 2022). In a large cross-sectional cohort study involving adults in community settings, anxiety symptoms were associated with worsened cognitive domains, especially processing speed and cognitive impairment, suggesting that anxiety can impact cognitive functioning by interfering with attention and working memory (Gulpers et al., 2022). A three-year prospective study found an association between anxiety symptoms and episodic memory decline in cognitively healthy older adults (Fung et al., 2018). Accelerating neurodegeneration through physiological mechanisms, anxiety symptoms could be a preclinical sign of cognitive impairment and an early sign of neurodegenerative diseases (Fung et al., 2018). Depression was also associated with a twofold increased risk of AD in a meta-analysis study (Sáiz-Vázquez et al., 2021). A study conducted during the COVID-19 pandemic also found a positive association between cognitive functioning and mental health outcomes, including anxiety and depressive symptoms, in the general population (Fiorenzato et al., 2021). Altogether, our findings underscore the necessity for future longitudinal studies to identify whether psychiatric symptoms are risk factors for impaired cognitive functioning in the general population globally and locally, considering the co-occurrence of depression and anxiety (Gulpers et al., 2022). We recommend further investigations regarding the impact of the pandemic on cognitive functioning and the relationship between cognitive functioning and mental health among the general population to design compelling interventions for remedial strategies during and after the COVID-19 pandemic. This study obtained a large sample that allows the identification of the most appropriate scale structure of the Chinese CFSS, and validation of the structure in an independent dataset.

### 4.3. Limitations

However, there are some limitations. First, this study applied a cross-sectional design and thus did not allow the assessment of test–retest reliability and responsiveness. Second, we collected data online to avoid social contact during the COVID-19 pandemic, and thus may not have reached individuals with limited internet access or who were illiterate, given the proportion of 17.9% of people aged 15 or above who were educated at a primary school level and below in 2020 (Census and Statistics Department, 2021b) and 92.9% coverage of mobile phones in 2021 in Hong Kong (Census and Statistics Department, 2022b). Third, we did not determine the optimal cutoff value of the CFSS to diagnose worsened cognitive functioning. Thus, future research on the screening function of the CFSS is warranted to identify vulnerable groups and provide them with tailored support. Fourth, the possible state of emotional negativity may bias the response to self-report measures of cognitive impairment (Watters and Williams, 2011). The negativity bias may obscure the identification of the scale structure of the Chinese CFSS. However, given the clear confirmation by the CFA, the potential influence should be minimal. Lastly, we did not examine the convergent validity of the Chinese CFSS with other self-reported or performance-based measurements including the HK-MoCA. Thus, more efforts to compare the results of the CFSS with another measure of cognitive functioning would be needed to strengthen the evidence regarding its validity.

## 5. Conclusion

In conclusion, the Chinese CFSS is a promising instrument for the self-reporting of perceived cognitive functioning. The strengths of this study are that it is useful and convenient to administer, as well as having adequate reliability and validity, which can be applied across ages, gender, and education levels. Owing to its self-reporting nature without healthcare professional assessment, it will be a potential screening tool for the general population for early identification of impaired cognitive functioning. More endeavors to evaluate the levels of cognitive functioning assessed using the CFSS scale and investigate the pandemic’s impact on cognitive functioning and its relationship with mental health among the general population are necessary to promote the public’s cognitive health. Furthermore, future research to investigate the factors associated with impaired cognitive functioning is warranted to help healthcare providers and policymakers devise compelling strategies and interventions aimed at preventing and managing cognitive impairment and disorders for public health and well-being in a timely and cost-effective manner.

## Data availability statement

The original contributions presented in the study are included in the article/supplementary material, further inquiries can be directed to the corresponding author/s.

## Ethics statement

The study was conducted in accordance with the principles of the Declaration of Helsinki and approved by the Institutional Review Board of the University of Hong Kong/Hospital Authority Hong Kong West Cluster (HKU/HA HKW IRB). The patients/participants provided their written informed consent to participate in this study.

## Author contributions

DF, JW, JK, HD, and LH: conceptualization. DF and KC: data curation and methodology. KC and SN: formal analysis. DF: supervision. KC, SN, and DF: writing – original draft. SL, HF, JW, JK, HD, LH, and MA: writing – review and editing. All authors who contributed to the manuscript have read and approved the submitted version of the manuscript.

## Funding

This study was supported by the Seed Funding for Basic Research of the University of Hong Kong (Grant number: 201910159243).

## Conflict of interest

The authors declare that the research was conducted in the absence of any commercial or financial relationships that could be construed as a potential conflict of interest.

## Publisher’s note

All claims expressed in this article are solely those of the authors and do not necessarily represent those of their affiliated organizations, or those of the publisher, the editors and the reviewers. Any product that may be evaluated in this article, or claim that may be made by its manufacturer, is not guaranteed or endorsed by the publisher.

## References

[ref1] American Educational Research Association; American Psychological Association; National Council on Measurement in Education (2014). Standards for Educational and Psychological Testing. Washington, DC: American Educational Research Association

[ref2] Annunziata M. A. Muzzatti B. Flaiban C. Giovannini L. Lucchini G. (2018). Cognitive functioning self-assessment scale (CFSS): further psychometric data. Appl. Neuropsychol. Adult 25, 1–4. doi: 10.1080/23279095.2016.122557510.1080/23279095.2016.1225575, PMID: 27645477

[ref3] Annunziata M. Muzzatti B. Giovannini L. Lucchini G. (2012). Cognitive functioning self- assessment scale (CFSS): preliminary psychometric data. Psychol. Health Med. 17, 207–212. doi: 10.1080/13548506.2011.596552, PMID: 21777093

[ref4] Bai W. Chen P. Cai H. Zhang Q. Su Z. Cheung T.. (2022). Worldwide prevalence of mild cognitive impairment among community dwellers aged 50 years and older: a meta-analysis and systematic review of epidemiology studies. Age Ageing 51:afac173. doi: 10.1093/ageing/afac173, PMID: 35977150

[ref5] Bradfield N. I. (2023). Mild cognitive impairment: diagnosis and subtypes. Clin. EEG Neurosci. 54, 4–11. doi: 10.1177/15500594211042708 34549629

[ref6] Census and Statistics Department. (2021a). 2021 Population Census. Available at: https://www.censtatd.gov.hk/en/scode600.html (Accessed February 2, 2023).

[ref7] Census and Statistics Department. (2021b). Hong Kong annual digest of statistics. Available at: https://www.censtatd.gov.hk/en/data/stat_report/product/B1010003/att/B10100032021AN21B0100.pdf (Accessed February 2, 2023).

[ref8] Census and Statistics Department. (2022a). Population estimates. Available at: https://www.censtatd.gov.hk/en/scode150.html#footer_map (Accessed February 2, 2023)

[ref9] Census and Statistics Department. (2022b). Thematic household survey report. Available at: https://www.censtatd.gov.hk/en/data/stat_report/product/B1130201/att/B11302752022XXXXB0100.pdf (Accessed February 2, 2023).

[ref10] Chiu H. (1994). Reliability and validity of the Cantonese version of mini-mental state examination-a preliminary study. J. Hong Kong Coll. Psychiatr. 4, 25–28.

[ref11] Costanza A. Amerio A. Aguglia A. Escelsior A. Serafini G. Berardelli I.. (2020). When sick brain and hopelessness meet: some aspects of suicidality in the neurological patient. CNS Neurol. Disord. Drug Targets 19, 257–263. doi: 10.2174/1871527319666200611130804, PMID: 32525785 PMC7569280

[ref12] Costanza A. Baertschi M. Weber K. Canuto A. (2015). Maladies neurologiques et suicide: de la neurobiologie au manque d’espoir [Neurological diseases and suicide: from neurobiology to hopelessness]. Rev. Med. Suisse 11, 402–405. doi: 10.1016/j.biopsycho.2021.108224, PMID: 25895218

[ref13] Costanza A. Xekardaki A. Kövari E. Gold G. Bouras C. Giannakopoulos P. (2012). Microvascular burden and Alzheimer-type lesions across the age spectrum. J. Alzheimers Dis. 32, 643–652. doi: 10.3233/JAD-2012-120835, PMID: 22842869

[ref14] Curran P. J. West S. G. Finch J. F. (1996). The robustness of test statistics to nonnormality and specification error in confirmatory factor analysis. Psychol. Methods 1, 16–29. doi: 10.1037/1082-989X.1.1.16

[ref15] Emmady P. D. Schoo C. Tadi P. (2022). “Major neurocognitive disorder (dementia)” in StatPearls (Treasure Island, FL: StatPearls Publishing), 7. 32491376

[ref16] Fiorenzato E. Zabberoni S. Costa A. Cona G. (2021). Cognitive and mental health changes and their vulnerability factors related to COVID-19 lockdown in Italy. PLoS One 16:e0246204. doi: 10.1371/journal.pone.024620410.1371/journal.pone.0246204, PMID: 33503055 PMC7840042

[ref17] Fung A. W. T. Lee J. S. W. Lee A. T. C. Lam L. C. W. (2018). Anxiety symptoms predicted decline in episodic memory in cognitively healthy older adults: a 3-year prospective study. Int. J. Geriatr. Psychiatry 33, 748–754. doi: 10.1002/gps.485010.1002/gps.4850, PMID: 29297937

[ref18] Gulpers B. J. Verhey F. R. Eussen S. J. Schram M. T. de Galan B. E. van Boxtel M. P.. (2022). Anxiety and cognitive functioning in the Maastricht study: a cross-sectional population study. J. Affect. Disord. 319, 570–579. doi: 10.1016/j.jad.2022.09.072, PMID: 36162695

[ref19] Hair JF William CB Barry JB Rolph EA. (2019). Multivariate Data Analysis. 8th Harlow: Cengage.

[ref20] Hooper D. Coughlan J. Mullen M. R. (2008). Structural equation modelling: guidelines for determining model fit. Electron. J. Bus. Res. Methods 6:5360.

[ref21] Hu L. T. Bentler P. M. (1999). Cutoff criteria for fit indexes in covariance structure analysis: conventional criteria versus new alternatives. Struct. Equ. Modeling 6, 1–55. doi: 10.1080/10705519909540118

[ref22] Hugo J. Ganguli M. (2014). Dementia and cognitive impairment: epidemiology, diagnosis, and treatment. Clin. Geriatr. Med. 30, 421–442. doi: 10.1016/j.cger.2014.04.001, PMID: 25037289 PMC4104432

[ref23] Jamieson A. Goodwill A. M. Termine M. Campbell S. Szoeke C. (2019). Depression related cerebral pathology and its relationship with cognitive functioning: a systematic review. J. Affect. Disord. 250, 410–418. doi: 10.1016/j.jad.2019.03.042, PMID: 30878653

[ref24] Jessen F. Amariglio R. E. Buckley R. F. (2020). The characterisation of subjective cognitive decline. Lancet Neurol. 19, 271–278. doi: 10.1016/S1474-4422(19)30368-0, PMID: 31958406 PMC7062546

[ref25] Kong A. P. H. Lam P. H. P. Ho D. W. L. Lau J. K. Humphreys G. W. Riddoch J.. (2016). The Hong Kong version of the Oxford cognitive screen (HK-OCS): validation study for Cantonese-speaking chronic stroke survivors. Neuropsychol. Dev. Cogn. B Aging Neuropsychol. Cogn. 23, 530–548. doi: 10.1080/13825585.2015.112732110.1080/13825585.2015.1127321 26702642

[ref26] Morris J. C. (2005). Mild cognitive impairment and preclinical Alzheimer’s disease. Geriatrics 60, 9–14. 16025770

[ref27] Muzzatti B. Cattaruzza N. Piccinin M. Flaiban C. Agostinelli G. Berretta M.. (2021). Cognitive function in long-term lymphoma survivors: relationship between subjective reports and objective assessments and with quality of life. Psychol Health and Med. 26, 968–979. doi: 10.1080/13548506.2020.177081510.1080/13548506.2020.1770815, PMID: 32459120

[ref28] Petersen R. C. (2004). Mild cognitive impairment as a diagnostic entity. J. Intern. Med. 256, 183–194. doi: 10.1111/j.1365-2796.2004.01388.x 15324362

[ref29] Pirani S. Kulhanek C. Wainwright K. Osman A. (2021). The reasons for living inventory for young adults (RFL-YA-II). Assessment 28, 942–954. doi: 10.1177/107319111990024210.1177/1073191119900242, PMID: 31976743 PMC8868000

[ref30] Sáiz-Vázquez O. Gracia-García P. Ubillos-Landa S. Puente-Martínez A. Casado-Yusta S. Olaya B.. (2021). Depression as a risk factor for Alzheimer's disease: a systematic review of longitudinal meta-analyses. J. Clin. Med. 10:1809. doi: 10.3390/jcm10091809, PMID: 33919227 PMC8122638

[ref31] Singham T. Saunders R. Brooker H. Creese B. Aarsland D. Hampshire A.. (2022). Are subtypes of affective symptoms differentially associated with change in cognition over time: a latent class analysis. J. Affect. Disord. 309, 437–445. doi: 10.1016/j.jad.2022.04.139, PMID: 35490883

[ref32] Streiner DL Norman GR Cairney J. (2015). Health Measurement Scales: A Practical Guide to Their Development and Use. 5th Oxford: Oxford University Press

[ref33] Strittmatter A. Sunde U. Zegners D. (2020). Life cycle patterns of cognitive performance over the long run. Proc. Natl. Acad. Sci. U. S. A. 117, 27255–27261. doi: 10.1073/pnas.200665311710.1073/pnas.2006653117, PMID: 33077581 PMC7959507

[ref34] Takemura N. Ho M. H. Cheung D. S. T. Lin C. C. (2022). Factors associated with perceived cognitive impairment in patients with advanced lung cancer: a cross-sectional analysis. Support Care Cancer 30, 9607–9614. doi: 10.1007/s00520-022-07377-9, PMID: 36178636

[ref35] Tong X. An D. McGonigal A. Park S. P. Zhou D. (2016). Validation of the generalized anxiety Disorder-7 (GAD-7) among Chinese people with epilepsy. Epilepsy Res. 120, 31–36. doi: 10.1016/j.eplepsyres.2015.11.019, PMID: 26709880

[ref36] University of California, Los Angeles. (2021). Confirmatory factor analysis in R with lavaan. Available at: https://stats.oarc.ucla.edu/r/seminars/rcfa/#s4c (Accessed February 2, 2023).

[ref37] Uwagbai O. Kalish V. B. (2022). “Vascular dementia” in StatPearls (Treasure Island, FL: StatPearls Publishing), 4–5.

[ref38] Watters A. J. Williams L. M. (2011). Negative biases and risk for depression; integrating self-report and emotion task markers. Depress. Anxiety 28, 703–718. doi: 10.1002/da.20854 21796742

[ref39] Wild D. Grove A. Martin M. Eremenco S. McElroy S. Verjee-Lorenz A.. (2005). Principles of good practice for the translation and cultural adaptation process for patient-reported outcomes (PRO) measures: report of the ISPOR task force for translation and cultural adaptation. Value Health 8, 94–104. doi: 10.1111/j.1524-4733.2005.04054.x10.1111/j.1524-4733.2005.04054.x, PMID: 15804318

[ref40] Wong B. P. S. Kwok T. C. Y. Chui K. C. M. Cheng T. S. T. Ho F. K. Y. Woo J. (2021). The impact of dementia daycare service cessation due to COVID-19 pandemic. Int. J. Geriatr. Psychiatry 37, 1–11. doi: 10.1002/gps.5621, PMID: 34490680 PMC8646460

[ref41] Wong A. Nyenhuis D. Black S. E. Law L. S. Lo E. S. Kwan P. W.. (2015). Montreal cognitive assessment 5-minute protocol is a brief, valid, reliable, and feasible cognitive screen for telephone administration. Stroke 46, 1059–1064. doi: 10.1161/STROKEAHA.114.00725310.1161/STROKEAHA.114.007253, PMID: 25700290 PMC4373962

[ref42] Wong A. Xiong Y. Y. Kwan P. W. Chan A. Y. Lam W. W. Wang K.. (2009). The validity, reliability and clinical utility of the Hong Kong Montreal cognitive assessment (HK-MoCA) in patients with cerebral small vessel disease. Dement. Geriatr. Cogn. Disord. 28, 81–87. doi: 10.1159/00023258910.1159/000232589, PMID: 19672065

[ref43] World Health Organization. (2022). Dementia. Available at: https://www.who.int/news-room/fact-sheets/detail/dementia (Accessed February 2, 2023).

[ref44] Yeung M. K. Lee T. L. Chan A. S. (2021). Depressive and anxiety symptoms are related to decreased lateral prefrontal cortex functioning during cognitive control in older people. Biol. Psychol. 166:108224. doi: 10.1016/j.biopsycho.2021.108224, PMID: 34785277

[ref45] Yu X. Tam W. W. Wong P. T. Lam T. H. Stewart S. M. (2012). The patient health Questionnaire-9 for measuring depressive symptoms among the general population in Hong Kong. Compr. Psychiatry 53, 95–102. doi: 10.1016/j.comppsych.2010.11.00210.1016/j.comppsych.2010.11.002, PMID: 21193179

